# Upgraded ESRF BM29 beamline for SAXS on macromolecules in solution

**DOI:** 10.1107/S0909049513010431

**Published:** 2013-05-18

**Authors:** Petra Pernot, Adam Round, Ray Barrett, Alejandro De Maria Antolinos, Alexandre Gobbo, Elspeth Gordon, Julien Huet, Jerôme Kieffer, Mario Lentini, Muriel Mattenet, Christian Morawe, Christoph Mueller-Dieckmann, Staffan Ohlsson, Werner Schmid, John Surr, Pascal Theveneau, Louiza Zerrad, Sean McSweeney

**Affiliations:** a ESRF, 6 Jules Horowitz, F-38043 Grenoble, France; b EMBL, 6 Jules Horowitz, F-38042 Grenoble, France

**Keywords:** small-angle X-ray scattering, proteins in solution, automation and high throughput, online HPLC, structural biology

## Abstract

Small-angle X-ray scattering (SAXS) measurements of proteins in solution are becoming increasingly popular with biochemists and structural biologists owing to the presence of dedicated high-throughput beamlines at synchrotron sources. As part of the ESRF Upgrade program a dedicated instrument for performing SAXS from biological macromolecules in solution (BioSAXS) has been installed at the renovated BM29 location. The optics hutch has been equipped with new optical components of which the two principal elements are a fixed-exit double multilayer monochromator and a 1.1 m-long toroidal mirror. These new dedicated optics give improved beam characteristics (compared with the previous set-up on ID14-3) regarding the energy tunability, flux and focusing at the detector plane leading to reduced parasitic scattering and an extended *s*-range. User experiments on the beamline have been successfully carried out since June 2012. A description of the new BioSAXS beamline and the set-up characteristics are presented together with examples of obtained data.

## Abstract 2

A description of the new ESRF BioSAXS beamline is given. The beamline presented is dedicated to small-angle X-ray scattering of macromolecules in solution operating with a high-throughput sample-changer robot and automated data analysis for quality control and feedback.

## 1. Introduction   

Beamline BM29 is the new dedicated station for small-angle X-ray scattering experiments on proteins in solution (BioSAXS) at the ESRF. As part of the ESRF Upgrade program (http://www.esrf.fr/about/upgrade), it replaces the previous fixed-energy beamline ID14-3 (Pernot *et al.*, 2010 ▶). The BioSAXS endstation is part of the UPBL10 (Upgrade Program BeamLine) complex and as an independent facility is in the direct vicinity of ID30 (MASSIF) and ID29 (MAD) macromolecular crystallography (MX) beamlines. Many of the components of the former ID14-3 experimental hutch equipment have been reused, as were the existing lead hutches at BM29 (previously operating as an EXAFS station). The significant changes were implemented in the new dedicated optics allowing energy tunability (7–15 keV), increased flux (up to 2 × 10^13^ photons s^−1^ as compared with 4 × 10^11^ photons s^−1^ achieved on ID14-3) and better beam focusing (beam size 500 µm × 500 µm or less) on the detector plane. These improved beam characteristics result in reduced parasitic scattering, shorter exposure times (by a factor of ten) and an extended *s*-range (0.02–6 nm^−1^, where scattering vector *s* = 4πsinΘ/λ) with respect to the previous endstation.

BM29 is a fully automated high-throughput beamline dedicated to proteins in solution which facilitates experiments for researchers. Similar high-throughput SAXS systems (Hura *et al.*, 2009 ▶; Martel *et al.*, 2012 ▶) have had significant impact in the structural biology community. Users are learning the ease of use of the beamline and a strong user community is already becoming established. In the past the reaction to SAXS by many experienced crystallographers was that SAXS experiments took too long, required too much effort and offered only little chance of success without expert guidance. Owing in part to the work of the SAXS group of EMBL Hamburg improving the software and the development by the trilateral collaboration between the EMBL Outstations in Grenoble and Hamburg and the ESRF of the automated sample handling and measurement system (in use from 2010), as well as a continuing program of training courses (supported by EMBO), these attitudes are changing. Data collection and cycle times (loading/unloading of sample and capillary cleaning) are short (about 1 min). Therefore, up to a thousand measurements can be undertaken per day, making experiment streamlining a crucial issue. A dedicated beamline control and data acquisition software, *BsxCuBE*, allows sample-changer control and real-time data display (two-dimensional and one-dimensional). It is connected to a data processing pipeline, driven by *EDNA* (Incardona *et al.*, 2009 ▶), providing automatic data processing up to *ab initio* models. Data collection parameters and results are logged and stored in the modified ISPyB database (a laboratory information management system that combines sample tracking and experiment reporting during synchrotron-based MX experiments; Delagenière *et al.*, 2011 ▶).

Since September 2012, a Malvern Gel Permeation Chromatography/Size-Exclusion Chromatography (GPC/SEC) system has been integrated into the beamline. A typical data collection using the size-exclusion column takes approximately 30 min (not counting the equilibration time of the column) but depends on the column and flow rate chosen. High-performance liquid chromatography (HPLC) allows improved data quality from complex systems such as membrane proteins or the measurement of individual states from dynamic equilibrium (which would otherwise be impossible). The use of HPLC can be interspersed with the sample-changer robot to optimize beam time.

## 2. Beamline overview   

**Figure 1:**
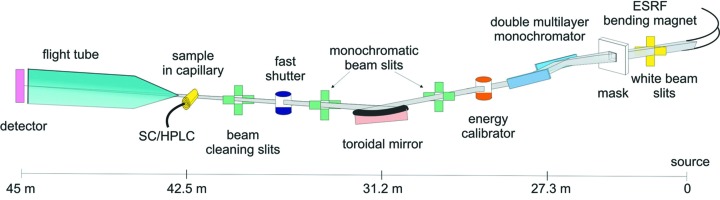
Layout of the BM29 BioSAXS beamline with the source-to-main-element distances.

Beamline BM29 is situated on a dipole (bending-magnet 29) with radiation centred at a −9 mrad horizontal angle which corresponds to a magnetic field of 0.85 T and a critical energy of 20.35 keV. The beam is defined by a water-cooled mask (opening 6 mm × 6 mm) located downstream of the white-beam slits (Fig. 1 ▶). The beamline layout and the majority of beamline components were designed by Pascal Theveneau. The retractable fluorescence screens, polycrystalline diamond for use in white beam, YAGs in monochromatic beam, with diodes installed downstream of each main optical element, allow easy beam alignment and diagnostics. The white-beam absorbers situated upstream reduce the low-energy power content of the beam impinging upon the multilayer (ML) monochromator and also decrease the low energy contamination of the diffracted beams. Beamline details are summarized in Table 1 ▶.

**Figure 2:**
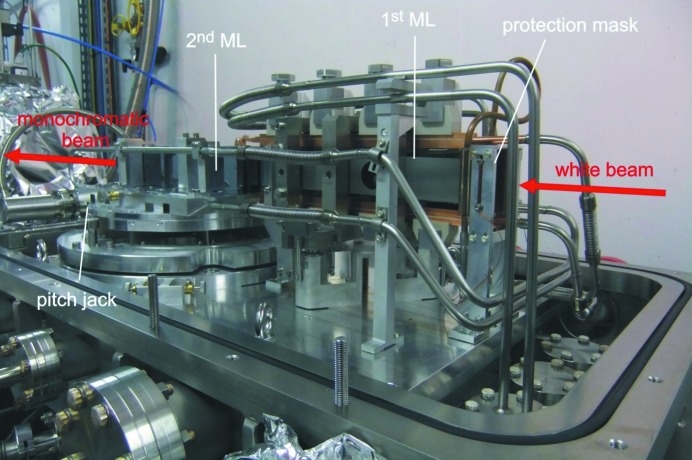
Inside view of the double ML monochromator vessel.

A water-cooled double ML monochromator was designed by Muriel Mattenet to a maximum absorbed power of 43 W at the first multilayer (Fig. 2 ▶). Two identical Ru/B_4_C ML coatings with 2.96 nm period were deposited in-house on two 300 mm-long Si substrates. The monochromator is a ‘nearly’ fixed-exit (beam moves by only 5 µm when changing energy from 7 to 15 keV) UHV-compatible device, scattering in the horizontal plane with a distance of 9 mm between the monochromatic and the incident white beams. A water-cooled beamstop is integrated into the monochromator vessel and serves to block any of the white beam escaping from the first ML but allows the monochromatic beam to pass. The size of the X-ray beam at the entrance of the monochromator is typically 4 mm × 4 mm. The energy is determined by rotation of the whole assembly by a given Bragg angle corrected by refraction (Morawe & Osterhoff, 2010 ▶); the axis of rotation coincides with the surface of the first ML. A fine rotation of the second ML is performed by a pushing jack if needed. No displacement of the second ML with respect to the first one is required as the footprint of the beam travels along the surfaces of both MLs. The ML coatings are designed to attenuate both second and third harmonics. Taking into account the reflectivity of the Rh-coated toroidal mirror located downstream, the total rejection of the harmonics is below 10^−4^ at 7 keV and below 10^−7^ at 15 keV. The energy calibration system installed just behind the monochromator can introduce into the beam metal foils of Fe, Cu and Pt, which have been chosen as they have absorption edges inside the accessible energy range (7.112, 8.98 and 11.564 keV, respectively).

The focusing element of the beamline is the 1.1 m-long cylindrical toroidal mirror reflecting vertically upwards at a glancing angle of 4 mrad. This Rh-coated mirror is located at 31.2 m from the source and focuses the monochromatic radiation in the detector plane, 13.5 m downstream. Typically, a 4 mm × 4 mm beam is focused to a spot of 0.5 mm × 0.5 mm in the detector plane, being 0.7 mm × 0.7 mm at the sample position (11 m from the mirror). The corresponding beam divergence is 130 µrad. The beam size in the sample plane and beam divergence can be further reduced, at the expense of photon flux, by decreasing the acceptance aperture of the focusing optic. The chromatic smearing effect upon the SAXS data due to the double ML bandwidth Δλ/λ of 1.5% is still negligible for typical measurements (Bolze *et al.*, 2002 ▶).

The experimental hutch equipment, *i.e.* slit box, sample stage, flight tube with motorized beamstop and the Pilatus 1M detector (Henrich *et al.*, 2009 ▶), was reinstalled (in the same configuration as on ID14-3) on a 4.5 m-long marble table of BM29. The maximum photon flux measured at 11 keV (using a calibrated Si diode) at the sample position is 2 × 10^13^ photons s^−1^ with a 4 mm × 4 mm white beam hitting the monochromator. To minimize the dose the samples are only irradiated during data collection using a fast experimental shutter (located 4 m upstream of the sample) to define the acquisition time. The transmitted intensity is monitored with a diode integrated in the beamstop and the intensity measured during data acquisition is used for normalization.

## 3. Ancillary facilities   

BM29 users have access to a sample preparation laboratory shared with ID29 visitors. Final sample preparation (dilutions, addition of ligands or altering the concentration of additives) and characterization can be performed immediately prior to the measurement and new samples prepared following feedback from data analysis. A nanodrop spectrophotometer, a cooled centrifuge, an ultrasonic thermal bath and some standard wet-lab equipment (balance, miliQ, ice machine, pipettes, *etc.*) are available for use. Access to other equipment is possible through the EMBL user laboratory by arrangement prior to the start of the experiment.

### 3.1. Sample-changer robot   

**Figure 3:**
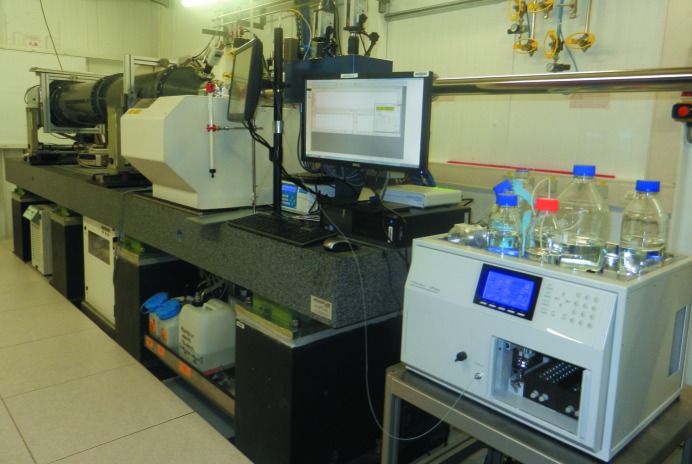
Experimental hutch set-up with HPLC and sample-changer units, slit box (blue), flight tube and detector at the back.

The beamline is equipped with a temperature-controlled automatic sample mounter developed in collaboration with the EMBL. A view of the experimental set-up in the BM29 experimental hutch including the sample changer is shown in Fig. 3 ▶. The robot has already been successfully implemented on ID14-3 and is described by Pernot *et al.* (2010 ▶). Since September 2010 the device has been in official user mode and has been highly appreciated by all visitors. The robot allows remote access and its software complexity/performance/user-friendliness is under continuous development. New features recently implemented are pipetting (transfer and mixing function) and an online spectrophotometer (allowing measurement of the sample concentration just prior to loading it into the capillary).

### 3.2. Beamline software   

**Figure 4:**
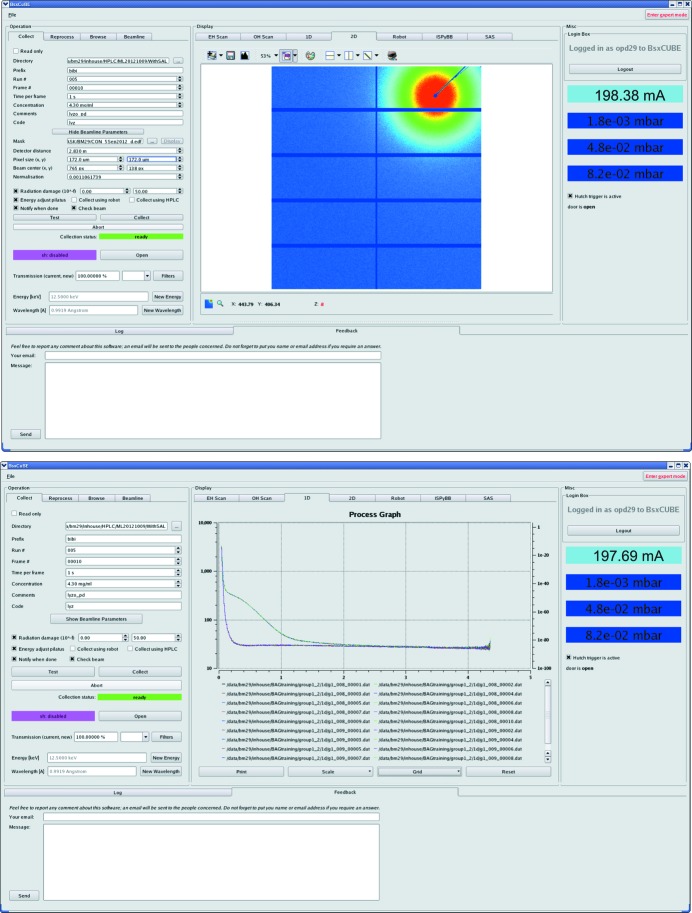
Data collection software *BsxCuBE* including the display of two-dimensional raw images and one-dimensional curves online.

Beamline control and data acquisition software, *BsxCuBE* (Biosaxs Customized Beamline Environment), has been developed to control the experiments and is shown in Fig. 4 ▶. *BsxCuBE* displays in real time recorded two-dimensional images as well as the processed one-dimensional curves. Data processing is performed online by three pipelines used subsequently and developed within the *EDNA* framework to provide the users with feedback and for quality control: (i) upstream data reduction built around *pyFAI* (python Fast Azimuthal Integration; Kieffer & Karkoulis, 2012 ▶) for data scaling and azimuthal integration, (ii) curve averaging taking into account radiation damage, background subtraction (matched buffer measured before and after each sample) and routine analysis on the resulting scattering curve, (iii) *ab initio* reconstructions.

Radiation damage is assessed by comparison of frames separated in time (typically ten frames per data collection). Frames varying by more than the calibrated threshold are excluded from the averaging and thus from any downstream processing. The output from the pipeline provides calculated parameters of the size (radius of gyration *R*_g_, estimated molecular mass and particle volume) plus an initial *ab initio* model for each construct measured as well as indicators of data quality to aid decision-making for subsequent data collection protocols. Results of the automated analysis are stored in a modified ISPyB database for SAXS. Crosschecks on the stored results give feedback regarding the need for repeat or extra measurements to complete the experiment. If required samples are available in the automated sample changer they can be measured; different concentrations can even be prepared with the pipetting function of the sample changer diluting the available sample with buffer. In addition, the full *ATSAS* software suite (Petoukhov *et al.*, 2007 ▶) is available on the beamline for manual data processing on a dedicated PC (high-power multicore CPU).

### 3.3. HPLC integration   

Proteins and especially complexes which are unstable can be difficult to measure and often require a final purification step immediately prior to measurement. For very dynamic systems the time available to perform a measurement after this purification is of the order of minutes. By performing purification online this allows a large number of systems to be investigated which would otherwise be impossible. HPLC is a common separation technique used routinely in many structural biology laboratories. The Malvern HPLC system (Viscotek RImax) is installed on the beamline and is integrated into the control and analysis software. Users bring their own compatible columns which are easily exchangeable (choice dependent on sample size). The column and UV measurement are located close to the sample exposure unit in order to minimize tubing. A simple and safe switch between sample-changer robot and the HPLC tubing is possible by a simple valve allowing normal robot mode while the column used is flushed by the corresponding buffer solution.

The HPLC system allows separation of aggregates and of different oligomeric species in mixtures in order to measure the individual parts. Measurement of membrane proteins can be undertaken with buffers containing detergent in order to correctly match the free detergent around the sample and in the background for accurate subtraction. Data processing automatically identifies peaks in the scattering and outputs model-independent parameters for all frames and an average of the matching frames from an individual peak. There are, in addition, sensing modules for measurement of the refractive index and light scattering giving independent concentration, molecular weight and hydrodynamic radius to provide extra quality control.

## 4. Facility access   

Access to the BioSAXS beamline is either through the BAG (Beamline Allocation Group) system used widely in the ESRF MX community or through a rolling application system which accepts proposals any time; if the peer-review is successful, beam time can be obtained within two months of its submission. There also exists a joint access for small-angle neutron scattering (SANS) users of Institut Laue–Langevin to enable complementary information provided by SANS (*i.e.* contrast variation) and SAXS to be obtained in a single trip to the Grenoble EPN campus (http://www.esrf.fr/UsersAndScience/UserGuide/Applying/ProposalGuidelines/MXnon-BAGproposal). The introduction of a liquid-handling robot, automated data collection and processing pipeline has made SAXS studies on biological macromolecules a high-throughput activity, and now also attracts interest from proprietary clients who can obtain beam time or their sample can be measured very quickly upon availability.

## 5. Data example   

**Figure 5:**
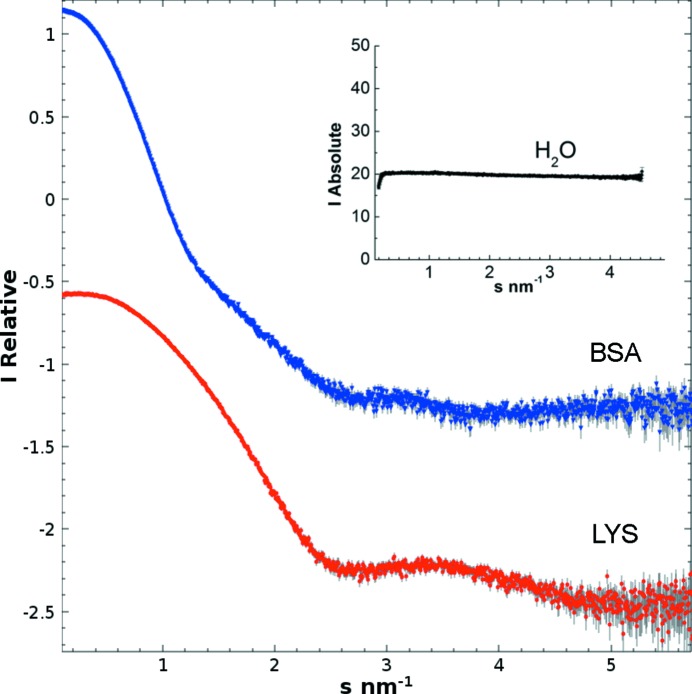
Buffer-subtracted experimental scattering pattern from bovine serum albumin (BSA) and lysozyme (LYS) scaled for visualization. The inset graph represents scattering from water (with empty capillary background subtracted), which was used for the absolute intensity calibration of sample curves.

Bovine serum albumin (BSA) and lysozyme (LYS) samples of 30 µl were exposed to X-rays while flowing through the 1.8 mm-diameter quartz capillary using the sample-changer robot for 10 s to avoid radiation damage. Fig. 5 ▶ shows the experimental scattering curves of BSA, LYS and water (inset) at a temperature of 293 K and a wavelength λ of 0.992 Å, as a function of scattering vector *s*. Data have been scaled to aid their visualization. The BSA averaged curve (from ten frames of 1 s exposure each) with buffer scattering subtracted (the average value of buffer alone measured before and after the sample measurement was applied) is a merge of 11.8 mg ml^−1^ and 2.2 mg ml^−1^ solutions. The LYS averaged curve corresponds to 8.6 mg ml^−1^ solution. Radii of gyration *R*_g_ of 3.02 nm and 1.4 nm, forward-scattered intensity at zero angle *I*(0) corresponding to a molecular weight of 72.1 kDa and 14.2 kDa were determined by Guinier analysis, for BSA and LYS, respectively. The molecular weights were obtained by scaling the curves to the scattering of water in units of kDa as described by Orthaber *et al.* (2000 ▶). The measured background on BM29 is lower than on ID14-3, thus enabling more reliable absolute calibration of scattering intensities. The data collection time is 10 s compared with 100 s at ID14-3, and gives comparable errors for the same concentration of protein.

## 6. Discussion and conclusions   

The newly constructed BM29 endstation has the capability to perform up to a thousand data collections on liquid protein samples per day in a completely automated manner. To ensure the BioSAXS facility at the ESRF remains competitive, the future requirements of users need to be anticipated. Solution SAXS experiments are usually required for systems which are either difficult to crystallize, produce or keep stable for longer periods of time. Therefore, to improve the feasibility of BioSAXS experiments, small sample volumes from ultra-dilute systems must be considered in the future. In order to take advantage of a small beam size with still elevated flux, the sample environment needs to be smaller to reduce the required volume. The first stage towards this is already accommodated in the current design by using a smaller diameter capillary tube (1 mm) in the existing exposure unit. Further reductions in size will be possible by the introduction of microfluidic devices (currently under development at the ESRF and other facilities).

There are currently 68 scientific publications reported from data collected at the former BioSAXS beamline ID14-3 after less than three years in operation. We expect the number of BioSAXS publications to increase rapidly with the data collected on BM29.

## Figures and Tables

**Table 1:** Beamline details

Beamline name	BM29
Source type	Bending magnet, centred at −9 mrad
Mirrors	1.1 m-long Rh-coated toroid, 4 mrad
Monochromator	Double multilayer, 2.96 nm spacing
Energy range (keV)	7–15
Wavelength range (Å)	0.82–1.77
Beam size (uncollimated) (µm)	4000 × 4000
Beam size (collimated, typical) (µm)	500 × 500 at detector plane
Flux (uncollimated) (photons s^−1^)	2 × 10^13^ (at 1.13 Å)
Flux (collimated, typical) (photons s^−1^)	1.3 × 10^13^ (at 1.13 Å)
Goniometer	Robot or HPLC
Cryo capability	277–333 K
Sample mounting	Quartz capillary
Detector type	CMOS hybrid pixel
Detector model	Pilatus 1M
2θ capabilities	0–4°
